# Maternal Vitamin and Mineral Supplementation and Rate of Maternal Weight Gain Affects Placental Expression of Energy Metabolism and Transport-Related Genes

**DOI:** 10.3390/genes12030385

**Published:** 2021-03-09

**Authors:** Wellison J. S. Diniz, Lawrence P. Reynolds, Pawel P. Borowicz, Alison K. Ward, Kevin K. Sedivec, Kacie L. McCarthy, Cierrah J. Kassetas, Friederike Baumgaertner, James D. Kirsch, Sheri T. Dorsam, Tammi L. Neville, J. Chris Forcherio, Ronald R. Scott, Joel S. Caton, Carl R. Dahlen

**Affiliations:** 1 Center for Nutrition and Pregnancy, Department of Animal Sciences, North Dakota State University, Fargo, ND 58102, USA; larry.reynolds@ndsu.edu (L.P.R.); pawel.borowicz@ndsu.edu (P.P.B.); alison.ward@ndsu.edu (A.K.W.); cierrah.kassetas@usda.gov (C.J.K.); friederike.baumgrtne@ndsu.edu (F.B.); james.kirsch@ndsu.edu (J.D.K.); sheri.dorsam@ndsu.edu (S.T.D.); tammi.neville@ndsu.edu (T.L.N.); joel.caton@ndsu.edu (J.S.C.); carl.dahlen@ndsu.edu (C.R.D.); 2 Central Grasslands Research and Extension Center, North Dakota State University, Streeter, ND 58483, USA; kevin.sedivec@ndsu.edu; 3 Department of Animal Science, University of Nebraska-Lincoln, Lincoln, NE 68583, USA; kacie.mccarthy@unl.edu; 4 Purina Animal Nutrition LLC, Gray Summit, MO 63039, USA; jforcherio@landolakes.com (J.C.F.); rrscott@landolakes.com (R.R.S.)

**Keywords:** caruncle, cotyledon, fetal programming, mineral, transcriptome, vitamin

## Abstract

Maternal nutrients are essential for proper fetal and placental development and function. However, the effects of vitamin and mineral supplementation under two rates of maternal weight gain on placental genome-wide gene expression have not been investigated so far. Furthermore, biological processes and pathways in the placenta that act in response to early maternal nutrition are yet to be elucidated. Herein, we examined the impact of maternal vitamin and mineral supplementation (from pre-breeding to day 83 post-breeding) and two rates of gain during the first 83 days of pregnancy on the gene expression of placental caruncles (CAR; maternal placenta) and cotyledons (COT; fetal placenta) of crossbred Angus beef heifers. We identified 267 unique differentially expressed genes (DEG). Among the DEGs from CAR, we identified *ACAT2, SREBF2*, and *HMGCCS1* that underlie the cholesterol biosynthesis pathway. Furthermore, the transcription factors *PAX2* and *PAX8* were over-represented in biological processes related to kidney organogenesis. The DEGs from COT included *SLC2A1*, *SLC2A3*, *SLC27A4*, and *INSIG1.* Our over-representation analysis retrieved biological processes related to nutrient transport and ion homeostasis, whereas the pathways included insulin secretion, PPAR signaling, and biosynthesis of amino acids. Vitamin and mineral supplementation and rate of gain were associated with changes in gene expression, biological processes, and KEGG pathways in beef cattle placental tissues.

## 1. Introduction

Maternal physiologic adaptation to pregnancy includes increased demand for nutrients to meet the maternal metabolic needs and nurture the developing fetus [1,2,3]. Furthermore, early gestation nutritional exposure affects the uterine environment and fetal development [4]. The fetomaternal interface provided by the placenta acts as a nutrient sensor to coordinate maternal nutrient supply and fetal metabolic requirements [5,6]. Thus, proper fetal development and nutrition are supported by an adequate nutrient supply through the placenta [7,8]. The placenta has many functions that include nutrient and waste product transport and hormone synthesis [6]. In ruminants, physiological exchanges between mother and fetus are supported by the caruncular-cotyledonary unit of the placenta, called the placentome [7]. While environmental stressors can trigger placental adaptations to meet fetal needs, these adjustments may affect fetal development and growth with long-lasting effects on metabolic function and performance [3,4,6,9,10].

Although growing evidence has shown the adverse effects of macronutrient (energy, protein, and fat) imbalances in fetoplacental development and function [11,12,13], less attention has been given to the role of micronutrients (vitamins and minerals) [4]. Vitamins and minerals play critical roles in animal health, growth, reproduction, and production, acting in structural, physiological, catalytic, and regulatory functions [14]. Maternal vitamin and mineral status has been suggested to affect hormonal regulatory pathways linking maternal metabolism with the fetoplacental unit [5,15]. Likewise, vitamins and minerals are required in biological processes such as energy metabolism, immune function, and gene expression [2,16,17]. Additionally, micronutrient-dependent enzymes that are involved with hormone synthesis and nutrient transport are present in the placenta [16]. While the placenta plays a pivotal role in mediating fetal needs, the changes and mechanisms associated with the placental adaptive responses to nutrient availability still need to be elucidated. Nonetheless, imprinted genes and differential gene expression are suggested as potential mechanisms that lead to placental plasticity [6].

Vitamins and minerals, as well as energy, protein, and fat are essential for proper fetal development and placental function. Lekatz et al. [18] reported hormonal and metabolic changes in pregnant ewes receiving supranutritional levels of selenium and/or nutrient restriction. Furthermore, changes in the expression of genes underlying angiogenesis and nutrient transport were identified in both caruncular and cotyledonary tissues [18]. In cows, myocardial necrosis and heart failure were identified in aborted selenium-deficient fetuses [19]. Maternal supplementation of organic trace minerals impacted offspring innate immune response and growth [20].

Despite the known effect of minerals on female reproductive traits and embryonic development [17], vitamin and mineral supplementation is still not widely adopted in cattle production [21]. Vitamins and minerals modulate the body’s energy homeostasis, and therefore, they are intricately related with the metabolism of carbohydrate, protein, and fat [14]. Furthermore, global nutrient restriction leads to reduced fetal growth and placental function [7]. Thus, a balanced diet that meets the requirements for macro and micronutrients is needed to optimize animal production. However, most studies focus on single nutrients without considering their multiple relationships on metabolic functions [22]. Similarly, the effects of vitamin and mineral supplementation on placental genome-wide gene expression have not yet been investigated.

Energy and protein availability are also essential for proper placental functions. Considering that nutrient requirements for first-calf heifers are greater than mature cows [23], it has been suggested that increasing nutrient supply through targeted supplementation strategies may optimize pregnancy rates, maternal-fetal transfer of nutrients, and increase offspring birth weights [24,25]. Herein, we tested the hypothesis that supplementing vitamin and minerals pre- and post-breeding to beef heifers grown at a low or moderate rate of maternal weight gain during the first trimester of gestation would affect placental gene expression. Therefore, we measured the gene expression profiles of maternal (caruncular; CAR) and fetal (cotyledonary; COT) portions of the placenta to identify differentially expressed genes (DEG), biological processes (BP), and pathways underlying placental development and function in response to early maternal nutrition. We identified 267 unique DEGs throughout all tissues and group contrasts. The over-represented functions are essential to fetal growth and development, and included fatty acid metabolism, hormone synthesis, nutrient transport, energy metabolism, and biosynthesis of amino acids.

## 2. Materials and Methods

All experiments and methods were performed following the relevant guidelines and regulations. The experimental design, animal management, and tissue collection were approved by the North Dakota State University Institutional Animal Care and Use Committee (IACUC A19012).

### 2.1. Animals, Experimental Design, and Tissue Collection

Angus-cross heifers (*n* = 35) were randomly assigned by initial body weight ($\bar{X}$ = 359.5 ± 7.1 kg) to a 2 × 2 factorial arrangement of treatments. The factors examined included vitamin and mineral supplementation (VTM or NoVTM) and rate of gain (low gain (LG) or moderate gain (MG)). The treatments were arranged as follows: (1) no vitamin and mineral supplementation and low gain (NoVTM_LG, *n* = 9); (2) vitamin and mineral supplementation and low gain (VTM_LG, *n* = 9); (3) no vitamin and mineral supplementation and moderate gain (NoVTM_MG, *n* = 9), and (4) vitamin and mineral supplementation and moderate gain (VTM_MG, *n* = 8).

Diets were delivered once daily via a total mixed ration and consisted of triticale hay, corn silage, modified distillers’ grains plus solubles, ground corn, and if indicated by treatment, mineral premix. To achieve MG (0.79 kg/d), heifers were fed the total mixed ration with the addition of the starch-based protein/energy supplement (a blend of ground corn, dried distillers’ grains plus solubles, wheat midds, fish oil, urea, and ethoxyquin). The LG heifers were maintained on the basal total mixed ration and targeted to gain 0.28 kg/d. Based on the National Research Council [26], the total mixed ration provided 105%, 158%, 215%, and 250% of the mineral requirements for NoVTM_LG, NoVTM_MG, VTM_LG, and VTM_MG treatments, respectively. Diet composition is described in Supplementary Materials, Table S1. The two rates of gain and VTM levels supplied to the heifers were chosen to represent two nutritional states (weight gain versus maintenance) as well as conditions applicable to beef production systems.

The VTM treatment started 71 to 148 days before artificial insemination by providing 0.45 kg/heifer daily of a ground corn and vitamin and mineral premix (113 g·heifer^−1^·d^−1^ of Purina Wind & Rain Storm All-Season 7.5 Complete, Land O’Lakes, Inc., Arden Hills, MN, USA). Based on the VTM starting date, heifers were assigned to one of seven breeding groups so that the supplementation period was at least 60 days for all. At breeding, heifers were randomly assigned to either LG or MG treatments within their respective VTM treatment. Heifers were bred by artificial insemination using female-sexed semen from a single sire. Pregnancy diagnosis was performed 35 days after artificial insemination, and fetal sex was determined on day 65 using transrectal ultrasonography. Further details of animal management were described elsewhere [27].

The VTM and rate of gain treatments were carried out until day 83 ± 0.27 of gestation, when uteroplacental tissues were collected through ovariohysterectomy [28]. The largest placentome closest to the fetus was collected and maternal (CAR) and fetal (COT) portions were manually dissected [29], snap-frozen, and stored at −80 °C.

### 2.2. Total RNA Isolation, Library Preparation, Sequencing, and Data Analysis

Total RNA of eight female samples per treatment was isolated from the CAR and COT tissues using the RNeasy^®^ kit (Qiagen^®^, Germantown, MA, USA) followed by on-column DNase treatment, according to the manufacturer’s protocol. Sample integrity and purity were evaluated using the Agilent 2100 Bioanalyzer and agarose gel electrophoresis. Strand-specific RNA libraries were prepared using the NEBNext^®^ Ultra™ II Directional RNA Library Prep Kit for Illumina (New England BioLabs^®^, Ipswich, MA, USA), and sequencing was carried out on the Illumina^®^ NovaSeq 600 platform. Library preparation and paired-end sequencing with 150-bp reads at a depth of 20 M reads/sample were carried out at Novogene Co. (Nanjing, China).

Sequencing adaptors, low-complexity reads, and reads containing low quality bases were removed in an initial data-filtering step. Reads with a PhredScore lower than 30 were filtered out. Quality control (QC) and read statistics were estimated with FastQC v0.11.8 [30] and MultiQC v1.9 [31] software. After QC, 29 and 31 samples (seven or eight samples per group) remained for further analyses from CAR and COT, respectively. Reads were mapped to the *Bos taurus* reference genome (ARS-UCD 1.2) [32] using the STAR aligner v. 2.7.3a [33]. Raw counts per gene were obtained using the –*quantMode GeneCounts* flag from STAR based on the gene annotation file (release 100, *Ensembl*). MultiQC, NOISeq [34], and edgeR [35] software were used to perform the post-mapping quality control.

### 2.3. Differential Expression and Functional Over-Representation Analyses

Genes with expression values lower than 1 count per million in 50% of the samples were filtered out. After filtering, the genes of CAR and COT tissues were analyzed using the DESeq2 v.1.22.1 R-package [36] to identify DEGs. The median of ratios method from DESeq2 was employed to normalize the data for sequencing depth and RNA composition [36]. The differential expression analysis used the negative binomial generalized linear model to fit gene expression level as a negative binomial distribution and Wald statistics to perform hypothesis testing [36]. The *svaseq* function of the R-package Surrogate Variable Analysis v.3.30.0 [37] was adopted to estimate unknown sources of variation in the RNA-Seq data. The DESEq2 model was used to measure the treatment effect while controlling for batch effect differences that included the surrogated variables and the heifer’s birthplace (farm of origin). To make all pair-wise comparisons between the four treatment groups, six contrasts were created as follows: (1) VTM_MG vs. NoVTM_LG, (2) VTM_MG vs. VTM_LG, (3) VTM_MG vs. NoVTM_MG, (4) VTM_LG vs. NoVTM_LG, (5) VTM_LG vs. NoVTM_MG, (6) NoVTM_MG vs. NoVTM_LG. Multiple testing adjustment of the *p*-values (*padj*) was performed using the Benjamini–Hochberg procedure for false discovery rate (FDR) [38]. Genes were identified as differentially expressed for each one of the contrasts when the false-discovery rate adjusted *p*-value (*padj*) cutoff ≤ 0.1 [11] and classified as up- or down-regulated based on the sign of the log2 fold change. The threshold (*padj* < 0.1) was defined a priori based on our experimental design. Furthermore, we used stringent quality control to remove lowly expressed genes and reduce the number of false-positive genes tested. As these are exploratory analyses, this combined approach allowed us to identify significant biological processes, while avoiding losing too much information.

Gene functional over-representation analysis was carried out using the ShinyGo v0.61 webtool [39] and the *B. taurus* annotation as background. This approach identified specific and common biological functions and KEGG pathways within and among gene lists for each tissue and contrast. Significant results after multiple testing adjustments were considered with an FDR ≤ 0.05.

**Figure 1:**
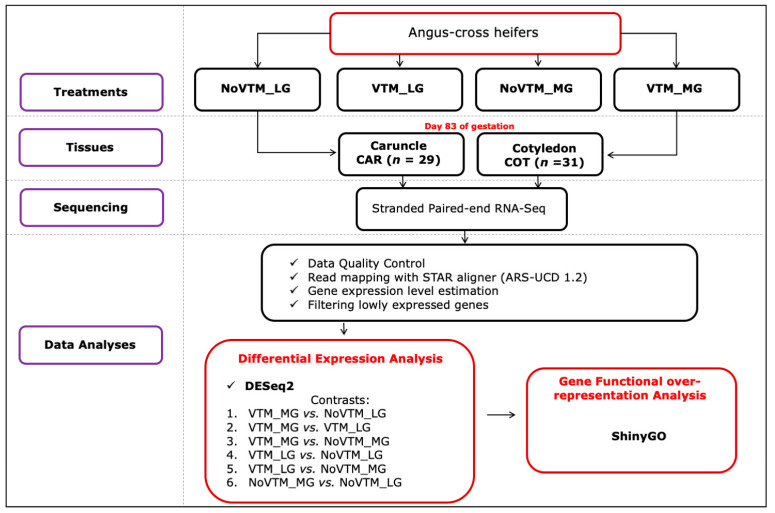
Experimental design and bioinformatics pipeline of the RNA-Seq-based differential expression analysis of bovine placental tissues—caruncle (CAR) and cotyledon (COT). The treatments were arranged as follows: NoVTM_LG—no vitamin and mineral supplementation and low gain; VTM_LG—vitamin and mineral supplementation and low gain; NoVTM_MG—no vitamin and mineral supplementation and moderate gain; and VTM_MG—vitamin and mineral supplementation and moderate gain.

An overview of the experimental design and data analyses pipeline is presented in Figure 1.

## 3. Results

We applied an RNA-Seq-based approach to identify differentially expressed genes in maternal (CAR) and fetal (COT) placental tissues of beef heifers subjected to vitamin and mineral supplementation and two rates of gain. On average, the sequencing of the tissues generated 22.7 M reads through the 60 samples with PhredScore > 30. The sequencing throughput and mapping rates per sample and tissue are reported in Table S2. On average, 97.0% and 96.2% of the reads from CAR and COT, respectively, were uniquely mapped to genes in the bovine reference genome (Table S2). After filtering, 13,252 genes from CAR and 12,795 from COT were analyzed to identify DEGs.

### 3.1. Differentially Expressed Genes

**Figure 2:**
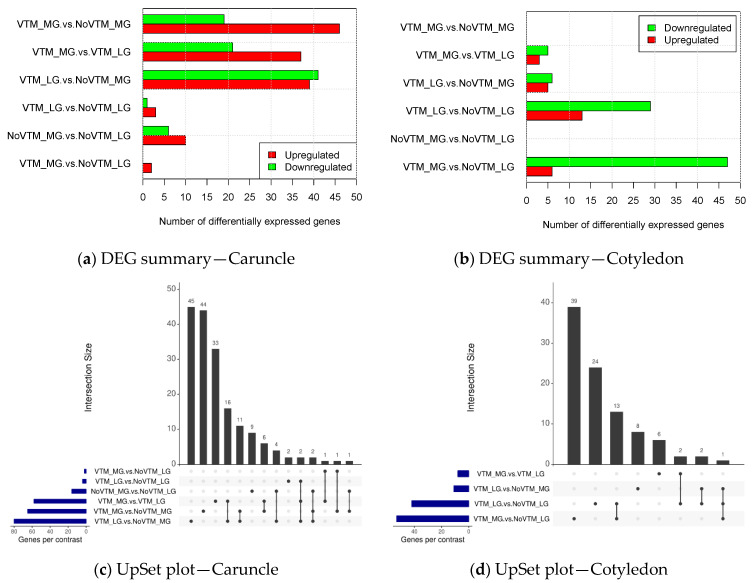
Differential gene expression summary of bovine placental tissues—caruncle (maternal placenta) (**a**) and cotyledon (fetal placenta) (**b**) (*padj* ≤ 0.1). The UpSet plot represents the intersection between the sets of differentially expressed genes (DEGs) from different contrasts of caruncle (**c**) and cotyledon (**d**). Each vertical bar shows the number of genes in the intersection. The dot plot reports the set participation in the intersection, and the horizontal bar graph reports the set sizes (total of DEGs). The treatments were arranged as follows: NoVTM_LG—no vitamin and mineral supplementation and low gain; VTM_LG—vitamin and mineral supplementation and low gain; NoVTM_MG—no vitamin and mineral supplementation and moderate gain; and VTM_MG—vitamin and mineral supplementation and moderate gain.

We identified 267 unique DEGs (*padj* ≤ 0.1) throughout all tissues and group comparisons. For the CAR tissue, gene expression analysis revealed 137 upregulated and 88 downregulated genes (Figure 2a), whereas in COT, 27 and 87 genes were upregulated or downregulated, respectively (Figure 2b). Our approach did not find significant DEGs for COT when comparing VTM_MG vs. NoVTM_MG and NoVTM_MG vs. NoVTM_LG.

The overlap between the sets of DEGs identified by the different contrasts are shown in Figure 2c,d. For CAR, we observed the greatest number of shared genes (*n* = 18) between the contrasts VTM_MG vs. VTM_LG and VTM_LG vs. NoVTM_MG. In the COT tissue, the VTM_LG vs. NoVTM_LG and VTM_MG vs. NoVTM_LG contrasts shared 14 genes between one another. When we compared the DEGs across tissues, most of them were tissue-specific, with only five genes shared between CAR and COT. The common DEGs between CAR and COT were: *DNMT3B*, *ESYT3*, *PRPFB1*, *FADS1*, and *TTC7A.* The DEGs are reported for each of the significant contrasts along with the fold-change values and annotation in Table S3 (CAR) and Table S4 (COT).

### 3.2. Functional Over-Representation Analysis

**Figure 3:**
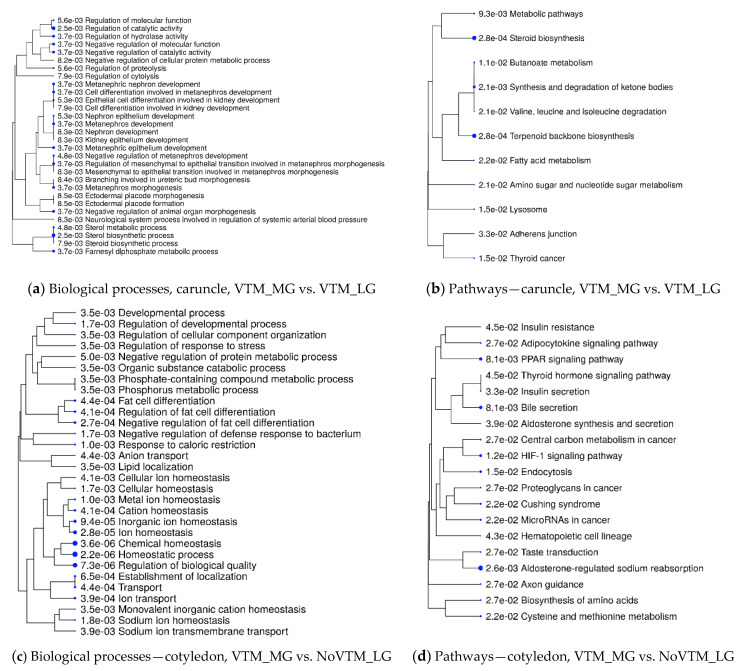
Functional over-representation analysis of differentially expressed genes (DEGs) from bovine placental tissues—caruncle (maternal placenta) and cotyledon (fetal placenta). Biological processes and KEGG pathways over-represented from DEGs in the caruncle (**a**,**b**) and cotyledon (**c**,**d**). The terms are hierarchically arranged based on functional similarity. The bigger the blue dot, the more significant the term is (FDR ≤ 0.05).

We retrieved significant biological processes (BP) and KEGG pathways by querying the DEGs of each contrast using the ShinyGO tool. Our approach identified 15 and 20 KEGG pathways from CAR and COT, respectively, that were over-represented by the DEGs (FDR ≤ 0.05). Likewise, 76 and 49 gene ontology BP terms were identified from CAR and COT, respectively (FDR ≤ 0.05). Figure 3 shows BP and KEGG pathways that were over-represented from DEGs of the VTM_MG vs. VTM_LG and VTM_MG vs. NoVTM_LG comparisons. The BP underlying the DEGs from CAR (Figure 3a) included, for example, regulation of molecular function and catalytic activity, organ morphogenesis and development (especially kidney and ureter). For CAR, among the over-represented pathways (Figure 3b) were the fatty acid metabolism, steroid biosynthesis, and terpenoid backbone biosynthesis. Due to the reduced number of DEGs, the contrasts NoVTM_MG vs. NoVTM_LG, VTM_MG vs. NoVTM_LG, and VTM_MG vs. NoVTM_LG from CAR did not retrieve any significantly enriched BP or KEGG pathways. Regarding COT, BP underlying the DEGs (Figure 3c) included metal ion homeostasis, ion transport, and regulation of developmental processes and response to stress, whereas the over-represented pathways (Figure 3d) included PPAR signaling, thyroid hormone signaling, adipocytokine signaling, insulin resistance, HIF-1 signaling, and cysteine and methionine metabolism. Biological processes and pathways identified for all the comparisons are provided in Tables S3 and S4.

## 4. Discussion

Nutrient demand increases throughout gestation to meet the requirements of the developing fetus. Growing evidence has shown that poor maternal nutrition, including vitamin and mineral deficiency, has adverse impacts on early placental development, with long-lasting effects on fetal programming of pre and postnatal growth and development [2,3,4,16]. In this study, we examined the impact of maternal vitamin and mineral supplementation (from pre-breeding to day 83) and two rates of gain (low or moderate) during the first 83 days of pregnancy on the gene expression of maternal (CAR) and fetal (COT) placental tissues. Our findings demonstrate that vitamin and mineral supplementation and rate of gain led to differential gene expression of CAR and COT tissues. However, the effect of rate of gain seems to be stronger on the maternal side, as for COT, few or no genes were identified as differentially expressed for the comparisons VTM_MG vs. VTM_LG and NoVTM_MG vs. NoVTM_LG. These findings suggest potential placental adaptations in response to maternal vitamin and mineral supplementation and rate of gain as indicated by the over-represented biological processes and pathways.

While our model achieved the targeted rates of gain (as designed), we did not find significant differences in fetal size or gravid uterine weight among the treatments [27]. On the other hand, fetal liver weight was greater (*p*-value = 0.05) from dams fed VTM than NoVTM [27]. Likewise, amino acid concentrations of maternal serum and allantoic and amniotic fluid in the same samples used in the current study were affected by vitamin and mineral supplementation and/or rate of gain [25]. Evidence suggests that biological mechanisms regulating normal growth, development, and nutrient utilization are programmed in utero for postnatal growth and adult function even during the earliest stages of development [40]. Additionally, large amounts of epidemiological data have shown that an impaired intrauterine environment has long-term consequences (reviewed in [4,41]). The key role performed by the placenta is mediated by changes in the gene expression that leads to differential programming of fetal tissues. Thus, imbalances in maternal vitamin and mineral availability during critical windows of development play a role in fetal tissue development as observed in fetal liver size.

Although no signs of vitamin and mineral deficiency or overload were observed in these pregnant beef heifers, changes in gene expression and concentrations of amino acids in fetal fluids suggest physiological adaptations to meet the fetal and maternal metabolic needs. Furthermore, the changes in gene expression of CAR seem to be greater than in COT as more DEGs were identified and were mainly upregulated. We know that changes in maternal metabolism are sensed by the placenta to meet fetal nutrient requirements based on the maternal resources available [5,42]. Thus, the dam may insulate the fetus against short-term nutrient imbalances by using their body reserves to sustain fetal growth [5]. Alternatively, the fetal placenta (COT) has specific homeostatic mechanisms to insulate the fetus, as suggested by the differential expression of glucose transporter genes.

### 4.1. Pathways Underlying Caruncular Differential Gene Expression

The placenta can adapt its capacity to supply nutrients in response to insults in the maternal-fetal environment [6]. Here, we found that vitamin and mineral supplementation combined with low or moderate gain affected pathways related to energy metabolism. Additionally, several BP and KEGG pathways underlying fatty acid metabolism, hormone biosynthesis, and amino acid degradation were identified in CAR. These are processes that require vitamins and minerals as structural components or enzymatic co-factors [14].

Underlying the fatty acid metabolism pathway, we identified *FADS1* and *ACAT2* as DEG for CAR. The *FADS1* gene codes for a rate-limiting enzyme involved with the metabolism and degradation of polyunsaturated fatty acids, such as docosahexaenoic acid and arachidonic acid [43]. Likewise, the protein encoded by *ACAT2* acts in lipid biosynthesis and regulates the synthesis of cholesteryl ester [44]. Additional genes related with cholesterol metabolism include *SREBF2*, which was upregulated in the VTM_LG vs. NoVTM_MG and downregulated in VTM_MG vs. VTM_LG in CAR. Sterol regulatory element-binding proteins (SREBPs) are transcription factors involved in cholesterol homeostasis and fatty acid uptake [45]. Steroid biosynthesis and sterol metabolic process were over-represented in our functional analysis of DEGs in CAR. Among the differentially expressed sterol-regulated genes underpinning cholesterol biosynthesis, we identified *HMGCS1*, *FDFT1*, *MSMO1*, and *SQLE* downregulated in VTM_MG vs. VTM_LG in CAR. Cholesterol is important for fetal development as a component in the cell membranes of the growing placenta and fetus [46]. Furthermore, cholesterol is the precursor of all steroid hormones, such as progesterone, that are required for normal gestation and fetal development [46]. The production of estrogens from cholesterol is supported by the enzymes present in the bovine trophoblast. According to Schuler et al. [47], the estrogen synthesized in the trophoblast suggests a role as local regulator of caruncular growth to produce a histotroph-like cell detritus. The histotroph in turn serves as an important source of nutrients for the fetus.

From the contrast between VTM_MG vs. NoVTM_MG, we identified the genes *CALM2*, *ATP2B4*, *CAMK2G*, and *BDKRB2* as over-represented in the calcium signaling and cyclic guanosine monophosphate (CGMP)-PKG signaling pathways. Calcium is not only essential for fetal development but also is an intracellular messenger that regulates, for example, gene transcription and cell proliferation [48]. Furthermore, calcium-mediated systems may activate steroidogenic activity of bovine placentomes [49]. The cGMP-PKG pathway plays a key role in vascular homeostasis and is mediated by nitric oxide and decreased calcium concentrations [50,51]. Previous studies have shown that maternal dietary treatments may impact placental vascularity and uterine blood flow [7,10,29]. Although we have not measured vascular development in the current study, we identified blood circulation, smooth muscle contraction, and circulatory system processes among the over-represented BP in CAR of VTM-supplemented heifers. Despite the lack of information regarding the role of vitamins and minerals in “driving” the increase in placental vascularity in bovine, Gernand et al. [16] reported that the human placenta is rich in micronutrient-dependent antioxidant enzymes that support normal maternal-fetal circulation. Moreover, vitamins E and D are suggested to enhance the expression of angiogenic factors in the placenta [52,53].

Interestingly, we identified several BP related to kidney morphogenesis, which were over-represented among the DEGs from the VTM_MG vs. VTM_LG contrast. The *PAX2* and *PAX8* genes were among DEGs in the BP such as kidney epithelium development and metanephros morphogenesis. These genes encode transcription factors that orchestrate kidney development, which is important for regulation of cardiovascular function, including blood pressure, later in life [54]. Additionally, the protein encoded by *PAX8* plays a key role in the development of other organs and tissues by interacting with the WT1 transcription factor, which has an essential role in the normal development of the urogenital system [55]. According to Christian et al. [15], changes in micronutrient availability may lead to hormonal adaptations, and consequently, affect kidney development and function. Moreover, Mao et al. [56] reported changes in the expression of placental genes that were involved with kidney function in mice fed high-fat or low-fat diets.

### 4.2. Pathways Underlying Cotyledonary Differential Gene Expression

The coordinated development and function between the CAR and COT placental tissues is responsible for providing the fetus with nutrients to support its metabolic demands [57]. Nonetheless, under nutritional stress, the placenta may increase the number and the surface area of cotyledons to improve the efficiency of placental transport [58]. These adjustments are not only related to placental vascular growth and angiogenesis [7,57] but also to the regulation of genes encoding for nutrient transporters [59]. As proposed by the placental nutrient sensing or fetal demand models [59,60], different mechanisms and placental responses underlie fetal-maternal nutrient cross-talk. Based on these models, maternal downregulation of genes encoding for nutrient transporters may lead to upregulation of fetal genes, and vice-versa, to balance maternal nutrient availability and fetal nutrient demand [59]. We observed that most of the nutrient transport DEGs from COT were classified as downregulated in the supplemented groups. In light of the above-mentioned models, this may suggest that the fetuses from supplemented dams met their nutritional requirements, whereas the non-supplemented fetuses optimized nutrient transport by upregulating gene expression. Furthermore, the rate of gain (i.e., maternal dietary intake to support a moderate or low rate of gain) seems to not affect COT gene expression under the conditions tested in the current study. According to Thayer et al. [5], the dam may homeostatically regulate macronutrient availability by mobilizing the maternal body’s reserve to supply the fetus. On the other hand, the authors argue that the body’s available store of micronutrients, such as vitamins and minerals, is limited, which may compromise their supply to the fetus when the maternal diet is micronutrient restricted [5].

Placental nutrient transporters are important for delivering nutrients such as glucose, amino acids, and fatty acids to the fetus [61]. Biological processes related to nutrient transport and ion transport were over-represented in our findings. Pathways related to insulin secretion and resistance, biosynthesis of amino acids, and PPAR signaling underlie the DEGs from the VTM_MG vs. NoVTM_LG groups in COT. Insulin is a potent hormone involved with energy metabolism and is essential for regulating glucose uptake and its serum levels [62]. Due to their role as cofactors in metabolic pathways, some minerals have been suggested to enhance insulin action [2]. For example, chromium improves glucose homeostasis through increased insulin sensitivity [63]. Glucose is the primary metabolic fuel for fetal metabolism, and it is crucial for fetal development [59]. Batistel et al. [64] reported that methionine supplementation during late gestation changed the expression profile of genes related to transport of amino acids, fatty acids, glucose, and vitamins of placentomes from dairy cows. Among the DEGs encoding glucose transporters, we identified *SLC2A1* (*GLUT1*) and *SLC2A3* (*GLUT3*). We also identified the *INSIG1* gene as differentially expressed. The protein encoded by *INSIG1* regulates cholesterol metabolism, lipogenesis, and glucose homeostasis. Furthermore, INSIG1 controls cholesterol synthesis through the SREBP and HMGCS1 proteins [65].

In addition to glucose, fatty acids are an important source of energy for placental function and fetal growth [66]. According to Lewis et al. [67], fatty acids are the precursors for PPAR transcription factors. PPARs are nuclear hormone receptors that are active in embryonic development and tissue differentiation through regulation of gene expression [66]. In the PPAR signaling pathway, we identified *ACSL3*, *SLC27A4*, and *PLIN2* as over-represented DEGs for COT from the contrast VTM_MG vs. NoVTM_LG. The *ACSL3* and *SLC27A4* genes encode proteins that are able to activate long chain fatty acids [68]. According to Nakahara et al. [69], the ACSL3 protein is involved with fatty acids uptake for synthesis of cellular lipids and degradation via beta-oxidation, while SLC27A4 acts in fatty acids transport [59]. The gene *PLIN2* is important for trophoblastic lipid droplet accumulation [70]. We identified BP related to lipid biosynthesis and metabolism among the DEGs from the VTM_MG vs. VTM_LG comparison as well. The DEGs play roles in sphingolipid biosynthesis (*DEGS2*) [71] and phospholipid biosynthesis and remodeling (*LPCAT1*) of the lipid droplets [72].

Among the DEGs, we found that *AARS1, IARS1, GARS1,* and *NARS2* were upregulated in the VTM_LG vs. NoVTM_LG comparison in COT. These genes were over-represented in the aminoacyl-tRNA biosynthesis pathway, and they encode key enzymes required for protein biosynthesis [73]. Furthermore, the *ARG2* and *MTR* genes were downregulated in the VTM_MG vs. NoVTM_LG comparison, and these genes are involved in the biosynthesis of amino acids. The *ARG2* protein is involved in the conversion of L-arginine into L-ornithine, which is a precursor to polyamines that support cell proliferation [74], whereas MTR catalyzes the final step in methionine biosynthesis [75]. Menezes et al. [25] reported increased concentrations of methionine and arginine in allantoic fluid in response to vitamin supplementation and a moderate rate of gain when evaluating the pregnant heifers used in the current study, which further supports the current findings.

## 5. Conclusions

By applying a genome-wide transcriptomic analysis, we identified genes differentially expressed from caruncular and cotyledonary placental tissues of pregnant heifers in response to maternal nutrition. Vitamin and mineral supplementation and low or moderate rate of gain are associated with changes in gene expression in placental tissues. Functional analysis of DEGs pointed out the pathways underlying energy metabolism, hormone synthesis, and nutrient transport. These findings shed light on the mechanisms via which maternal nutrition may regulate placental function and, potentially, fetal growth and development. Furthermore, our findings, for the first time, unravel the putative placental adaptations in response to maternal vitamin and mineral supplementation from pre-breeding through to the first trimester (until day 83) of gestation.

## Data Availability

All relevant data are within the paper and its Supplementary Information files. All sequencing data is publicly available on NCBI’s Gene Expression Omnibus (https://www.ncbi.nlm.nih.gov/geo/query/acc.cgi?acc=GSE165378).

## Supplementary Materials

The following are available online at https://www.mdpi.com/2073-4425/12/3/385/s1, Table S1: Nutrient composition of total mixed ration and supplements provided to beef heifers; Table S2: Caruncular and cotyledonary RNA sequencing summary and mapping statistics; Table S3: Supplementary information related to the differentially expressed genes, biological processes, and pathways from caruncular tissue; Table S4: Supplementary information related to the differentially expressed genes, biological processes, and pathways from cotyledonary tissue.
